# Longer Disciplined Tapers Improve Marathon Performance for Recreational Runners

**DOI:** 10.3389/fspor.2021.735220

**Published:** 2021-09-28

**Authors:** Barry Smyth, Aonghus Lawlor

**Affiliations:** Insight SFI Research Centre for Data Analytics, University College Dublin, Dublin, Ireland

**Keywords:** marathon taper, data analysis, marathon training, recreational runners, marathon performance

## Abstract

For marathoners the taper refers to a period of reduced training load in the weeks before race-day. It helps runners to recover from the stresses of weeks of high-volume, high-intensity training to enhance race-day performance. The aim of this study was to analyse the taper strategies of recreational runners to determine whether particular forms of taper were more or less favorable to race-day performance.

**Methods:** We analyzed the training activities of more than 158,000 recreational marathon runners to define tapers based on a decrease in training volume (weekly distance). We identified different types of taper based on a combination of duration (1–4 weeks of decreasing training) and discipline (strict tapers progressively decrease training in the weeks before the marathon, relaxed tapers do not) and we grouped runners based on their taper type to determine the popularity of different types of taper and their associated performance characteristics.

**Results:** Kruskal-Wallis tests (H(7)≥ 521.11, *p* < 0.001), followed by posthoc Dunns tests with a Bonferroni correction, confirmed that strict tapers were associated with better marathon performance than relaxed tapers (*p* < 0.001) and that longer tapers of up to 3 weeks were associated with better performance than shorter tapers (*p* < 0.001). Results indicated that strict 3-week tapers were associated with superior marathon finish-time benefits (a median finish-time saving of 5 min 32.4 s or 2.6%) compared with a minimal taper (*p* < 0.001). We further found that female runners were associated with greater finish-time benefits than men, for a given taper type ( ≤ 3-weeks in duration), based on Mann Whitney U tests of significance with *p* < 0.001.

**Conclusion:** The findings of this study for recreational runners are consistent with related studies on highly-trained athletes, where disciplined tapers were associated with comparable performance benefits. The findings also highlight how most recreational runners (64%) adopt less disciplined (2-week and 3-week) tapers and suggest that shifting to a more disciplined taper strategy could improve performance relative to the benefits of a less disciplined taper.

## Introduction

The taper refers to a gradual reduction in training load for athletes in the days or weeks before a competitive event (Houmard, 1991; Banister et al., 1999; Mujika and Padilla, 2003; Wilson and Wilson, 2008; Mujika, 2009; Le Meur et al., 2012). Its main aim is to help maximize the physiological adaptations that arise from training by providing athletes with an opportunity to recover from the considerable fatigue that accumulates during training. Research has shown that carefully controlled tapers can lead to significant performance benefits for athletes in a variety of sporting disciplines (Neary et al., 1992; Banister et al., 1999; Berger et al., 1999; Bosquet et al., 2007; Grivas, 2018; Skovgaard et al., 2018).

A taper can be implemented in several ways, for example, by adjusting training frequency, volume and/or intensity (Wenger and Bell, 1986; McConell et al., 1993) according to a prescribed schedule over a pre-determined period of time (Houmard, 1991; Mujika, 1998; Mujika and Padilla, 2003; Spilsbury et al., 2015). However, great care is required to strike the right balance between maximizing performance, overtraining (Morgan et al., 1987; Berger et al., 1999; Halson and Jeukendrup, 2004) and de-training (Fleck, 1994; Mujika and Padilla, 2000). Short tapers may not be sufficient to elicit the necessary recovery response that is required for optimal performance (Morgan et al., 1987), whereas tapers that are too long, or where training load is reduced by too much or too quickly, could lead to a de-training effect (Houmard et al., 1989; Mujika and Padilla, 2000), which could also compromise performance.

To date, most of the research on tapering has focused on small numbers of disciplined, well-trained or competitive athletes, who often benefit from personalized training programmes and/or individual coaching, with carefully controlled taper protocols implemented before competition. Much less is known about the tapering habits of recreational athletes, although the conventional wisdom is that tapering can be beneficial for athletes of all abilities before a competitive event. The main aim of this work is to analyze tapering patterns, and their performance effects, among 158,117 recreational runners (almost 126,000 men and >32,000 women), using activity data collected by the popular Strava training app, and thus highlighting the analysis opportunity that exists for large-scale, real-world data collected by apps such as Strava^1^, RunKeeper^2^, and MapMyRun^3^. The main research questions that we wish to answer include:

- *RQ1*: How can we distinguish between different types of tapers that are common among recreational runners, and how do they vary in terms of their adoption rates and characteristics (duration, training decrease etc.)?
- *RQ2*: How does the type of taper impact marathon performance? Are certain types of tapers associated with finish-time costs or benefits when we control for runner ability?
- *RQ3*: How does taper performance depend on the sex of runners? Do male or female runners enjoy more favorable taper benefits?

To answer these questions we will present a framework for distinguishing between different types of taper, based on a reduction in training volume (weekly distance) in the 4-week period before a marathon. Tapers are classified based on their duration—the number of weeks before race-day when training begins to be reduced—and discipline—the consistency of the training reductions in the weeks before a race—to distinguish between 1, 2, 3, and 4-week tapers and strict vs. relaxed tapers. Runners are grouped according to the taper type to compare their race-day performance.

## Materials and Methods

### Subjects and Data

The data set used in this study is based on an anonymous data set of running activities made available to the authors by Strava Inc., as a part of a data-sharing agreement. The data set includes 158,117 unique runners who completed a marathon between 2014 and 2017. Marathon races were identified by matching the start dates and locations of known marathon races with matching clusters of runners undertaking 42.2 km activities. Each runner is associated with an average of approximately 23 weeks of training data (just over 3.5 activities per week on average) before their marathon race, plus the marathon activity itself. If a runner completed more than one marathon in a given year, then only their fastest marathon (and its training activities) was selected for inclusion in the data set. The main reason for this is to focus on a runner's goal-race each year and to avoid overlapping training periods when runners complete multiple marathons in a single year; see section Implications for Recreational Runners for further discussion. Table 1 summarizes the key characteristics of the male and female runners in this data set.

**Table 1:** Summary data set statistics (μ ± σ) for male and female runners.

	**Female**	**Male**
Total runners	32,163	125,954
Number of training weeks	23.35 ± 5.08	23.83 ± 4.68
Mean activities/week	3.57 ± 1.50	3.59 ± 1.66
Mean weekly distance (km)	39.29 ± 15.46	43.16 ± 18.04
Mean Weekly Pace (min/km)	6.15 ± 0.83	5.52 ± 0.70
Mean fastest 10 km pace (min/km)	4.87 ± 0.96	4.28 ± 0.77
Marathon finish-time (h:min:s ± min:s)	4:09:43.8 ± 43:7.2	3:41:36 ± 41:9

Each individual activity session is composed of the raw data collected by (or synchronized with) Strava during training. This raw data consists of a time-series of distance values indicating how far an athlete ran in a given period of time. Since different runners use different devices to track their data (smartwatches, mobile phones, etc.) the sampling frequency and accuracy of these time series can vary. For this work, these raw time series are transformed and smoothed to produce a sequence of pacing values (min/km) representing the mean pace at 100 m intervals during an activity; thus a 10 km activity will contain a sequence of 100 pacing values. These data are then combined into a weekly representation by aggregating each week's activities to calculate key features such as weekly distance, mean/fastest weekly pace, number of activities per week etc.

### Defining the Taper

Most recreational marathon runners are not as disciplined as highly-trained, competitive or elite runners, which often leads to less organized tapers. For example, highly-training, competitive athletes tend to follow a carefully controlled taper by progressively reducing training volume in the weeks before a race. In contrast, approximately two-thirds of recreational runners punctuate their decreasing taper weeks with a week of increasing training volume. This makes it challenging to characterize recreational tapers using a conventional taper framework (Mujika and Padilla, 2003). Therefore, in this study we propose a modified taper framework that is better suited to the recreational runners in our data set. We define a taper in terms of decreases in weekly training volume (total weekly distance) during the *taper period* relative to a *baseline period* as follows:

- The *taper period* refers to the 4 weeks directly before race day. These are the weeks considered to be part of a runner's taper even though not every runner will taper for the full 4-week period. In our data set all runners have logged some activities during at least three of the four taper weeks.
- The *baseline period* refers to weeks 5 and 6 before race day (*i* = 5, 6 in Equation 1). These weeks are used to calculate a mean baseline weekly distance (*D*_*baseline*_) for a runner *r* (see Equation 1) against which to judge changes in weekly distance during the taper period.
- The relative change in weekly distance during the taper period, the *taper degree*, is calculated according to Equation 2 for the first taper period week (4-weeks from race-day) and with Equation 3 for the remaining 3 weeks.

Thus, if a runner's mean weekly distance during weeks 5 and 6 is 60 km and this decreases to 50 and 40 km in weeks 4 and 3, respectively, then the taper degrees during these weeks will be –0.167 and –0.20, respectively. These are known as *down* weeks because weekly distance decreases; if weekly distance increases then that week is an *up* week. We define a runner's *taper profile* as the sequence of *taper degrees* during the taper period; thus a taper profile such as (0.0, –0.1, –0.2, –0.3) indicates a runner whose weekly distance was stable during week 4 before the race, but then decreased progressively for the next three weeks, by 10, 20, and 30%, week on week, respectively.


(1) $$\begin{array}{r} D_{baseline}\left(r\right)=\frac{\underset{i=5,6}{\sum }D_{weekly}\left(r,i\right)}{2} \end{array}$$

(2) $$\begin{array}{r} degree\left(r,4\right)=\frac{D_{weekly}\left(r,4\right)-D_{baseline}\left(r\right)}{D_{baseline}\left(r\right)} \end{array}$$

(3) $$\begin{array}{r} degree\left(r,i\right)=\frac{D_{weekly}\left(r,i\right)-D_{weekly}\left(r,i+1\right)}{D_{weekly}\left(r,i+1\right)} \end{array}$$

We categorize different types of taper based on duration and discipline. The taper duration (see Equation 4) refers to the number of *down* weeks during the 4-week taper period; thus, a 2-week taper refers to a runner whose training volume decreased during only two of the four taper weeks. The taper discipline refers to how the down weeks are distributed during the taper period. An *n*-week taper is strict if its *n* down weeks occur consecutively and directly before race-day, otherwise it is a relaxed taper; see Equations 5 and 6.

In this way, a taper profile of (+0.1, –0.2, –0.1, –0.3) corresponds to a strict 3-week taper, because the 3 down weeks are consecutive and immediately before race-day; note that in a strict taper it is not necessary for the degree of the down weeks to be increasingly negative during the taper. In contrast, a profile such as (-0.1, +0.2, +0.1, -0.3) corresponds to a relaxed 2-week taper, because it has two down weeks, but these weeks are not the 2 weeks immediately before race day. Thus, in strict tapers, down weeks are never followed by up weeks, but in relaxed tapers at least one down week is followed by an up week. This means that a taper profile such as (–0.2, +0.1, –0.3, –0.4) is characterized as a relaxed 3-week taper—because there are three down weeks but one is followed by an up week—rather than a strict 2-week taper; even though this profile has two consecutive down weeks immediately before race-day there is also a third down week 4 weeks before race day.


(4) $$\begin{array}{r} duration\left(r\right)=\underset{1\leq i\leq 4}{\sum }degree\left(r,i\right)<0 \end{array}$$

(5) $$\begin{array}{r} strict\left(r,n\right)\Leftrightarrow \left(\underset{1\leq i\leq n}{\sum }degree\left(r,i\right)<0\right)=n \end{array}$$

(6) $$\begin{array}{r} relaxed\left(r,n\right)\Leftrightarrow \left(duration\left(r\right)=n\right)\;\wedge \;\neg strict\left(r,n\right) \end{array}$$

Accordingly, we can define eight mutually exclusive types of taper: there are strict and relaxed forms of 1-, 2-, and 3-week tapers, but only strict 4-week tapers are feasible. And if a taper profile does not correspond to one of these, because it has no down weeks, then it is a *non-taper*, in the sense that it shows no decrease in training volume during the taper period. In this way, it is straightforward to assign a runner to a unique taper type based on their taper profile.

### Performance Metrics

We use three different metrics to evaluate race performance. The most straightforward is finish-time (*FT*), which is the marathon-time of a runner, measured in minutes. One shortcoming of this measure is that it could be misleading if, for example, faster runners tend to dominate a particular type of taper, making it look like this taper leads to faster finish-times. For this reason we also calculate the so-called finish-time *efficiency* (*FTE*), which measures how close a runner's marathon race-pace is to the fastest 10 km pace they achieved during training; see Equation 8. An FTE of 85% means a runner's marathon pace was 15% slower than their fastest 10 km training pace. This metric is intended to capture their marathon performance as a function of their ability, and offers a way to normalize performance with respect to runner ability. A higher FTE means that a runner was able to maintain a marathon pace that was closer to their fastest 10 km pace, compared with a lower FTE.


(7) $$\begin{array}{r} mrp\left(r\right)=\frac{FT\left(r\right)}{42.195} \end{array}$$

(8) $$\begin{array}{r} FTE\left(r\right)=\left(\frac{fastest_{10km}\left(r\right)}{mrp\left(r\right)}\right)\times 100 \end{array}$$

As a third performance metric, we also calculate the *finish-time benefit* (*FTB*) of a particular type of taper, as an estimate of how many finish-time minutes are gained or lost in the marathon due to a given taper type. It is not obvious how to do this, since we cannot readily compare the finish-times of runners based on two different tapers. However, we can calculate a useful estimate as follows. First, we measure finish-time benefit relative to a relaxed 1-week taper, using this taper as a *minimal taper baseline*; we don't use non-tapers as a baseline because they are exceedingly rare and so not representative as a common tapering practice. Next, we calculate the median FTE for these relaxed 1-week tapers (*T*1_*R*_) as a baseline FTE. The assumption is that FTE is likely to suffer relative to longer or more strict tapers and we refer to this median as the *untapered* FTE or *uFTE* as shown in Equation 9.


(9) $$\begin{array}{r} uFTE=median\left(FTE\left(r\right)\right)\;:\;\forall r\in T1_{R} \end{array}$$

Then, we use *uFTE* to estimate an *expected finish-time* (eFT) for differently tapered runners, based on their fastest 10 km pace as in Equation 10. Effectively this is the finish-time that a runner might be expected to achieve after a relaxed 1-week taper, based on their fastest 10 km training pace and an assumed untapered FTE.


(10) $$\begin{array}{r} eFT\left(r\right)=fastest_{10km}\left(r\right)\times \frac{100}{uFTE\left(r\right)}\times 42.195 \end{array}$$

Finally, we calculate the finish-time benefit based on the difference between the expected and actual finish-times of runners, as shown in Equation 11 (FTB); Equation 12 also calculates the finish-time benefit as a percentage of finish-time. A positive FTB means that a runner's actual finish-time is *faster* than their expected (untapered) finish-time. Thus, we argue that the FTB metric provides an objective, normalized estimate of relative taper performance.


(11) $$\begin{array}{r} FTB\left(r\right)=eFT\left(r\right)-FT\left(r\right) \end{array}$$

(12) $$\begin{array}{r} \%FTB\left(r\right)=100\times \frac{FTB\left(r\right)}{FT\left(r\right)} \end{array}$$

### Statistical Analysis

In the main analysis we calculate the *FT*, *FTE*, *FTB* metrics based on taper type to explore whether different forms of taper (long vs. short, strict vs. relaxed) are associated with improved marathon performance. Since the values of these metrics are not normally distributed we use Kruskal Wallis tests to determine whether taper type has a statistically significant effect on performance, followed by posthoc Dunn's tests to identify which pairs of taper types are significantly different, if any, using a significance level of *p* < 0.05.

We also consider the effect of runner sex on performance to determine whether the benefits of tapers differ for male and female runners by calculating the finish-time benefits as a percentage of marathon finish-times, and using a Mann Whitney U test to compare these for male and female runners by taper type.

Finally, we conduct a cross-sectional regression analysis in order to determine the effect of gender and ability on marathon finish-time. The model is specified in Equation 13, where *FT*_*i*_ denotes the finish-time of runner *i*, *taper*_1_..*taper*_7_ are dummy variables corresponding to each taper type, including the non-taper but excluding the relaxed 1-week taper which is used as the baseline, *Male* is a dummy variable to indicate that the runner is male, *FastestPace* is the runner's fastest 10 km pace during training, and *e*_*i*_ is a stochastic error term representing the unexplained factors that affect finish-time.


(13) $$\begin{array}{r} FT_{i}=c+\left(\sum_{1\leq i\leq 7}\beta_{i}\times taper_{i}\right)+\beta_{8}\times Male+\beta_{9}\times FastestPace+e_{i} \end{array}$$

## Results

**Figure 1:**
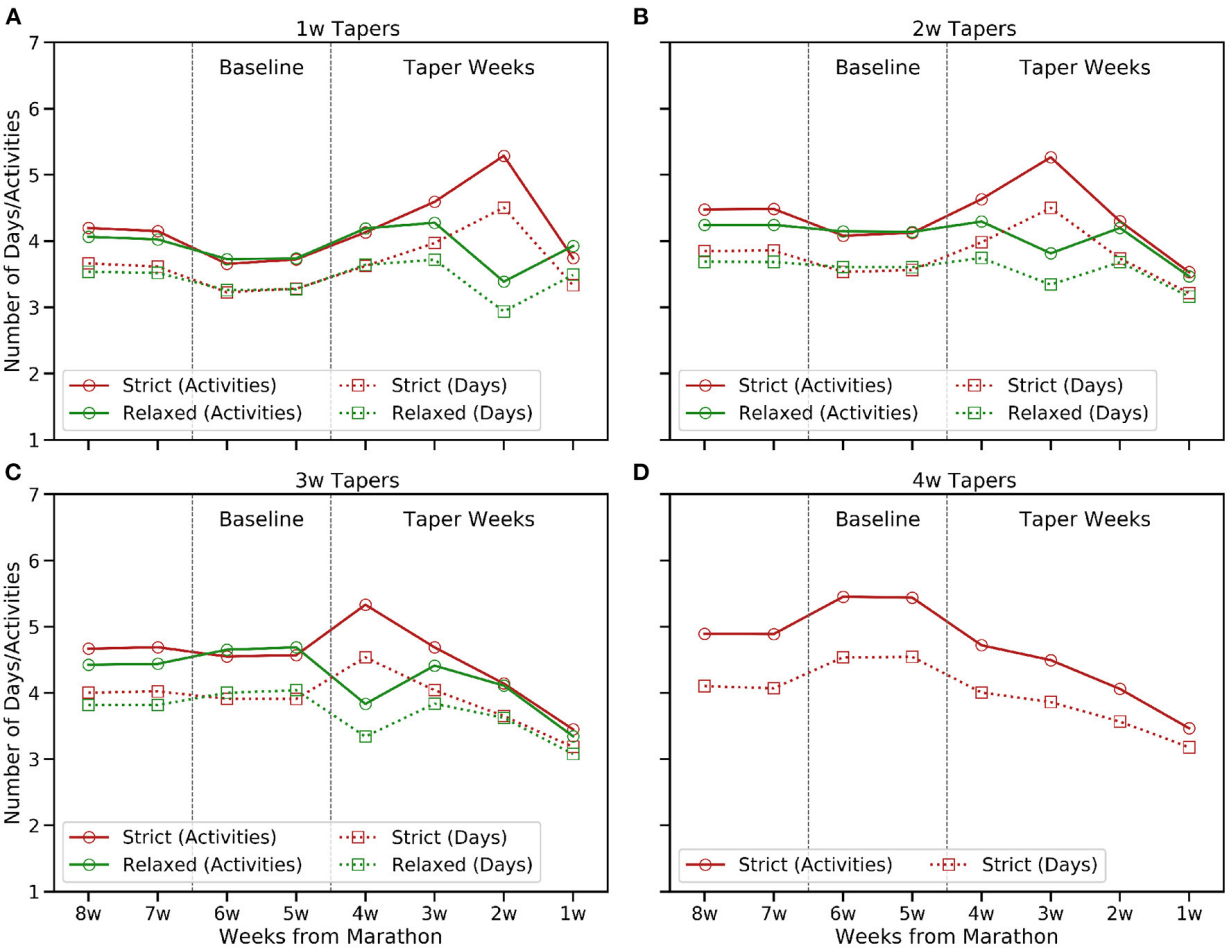
The mean number of weekly activities and active days in the 8 weeks before race-day by taper type. **(A)** 1w, **(B)** 2w, **(C)** 3w, and **(D)** 4w tapers.

**Figure 2:**
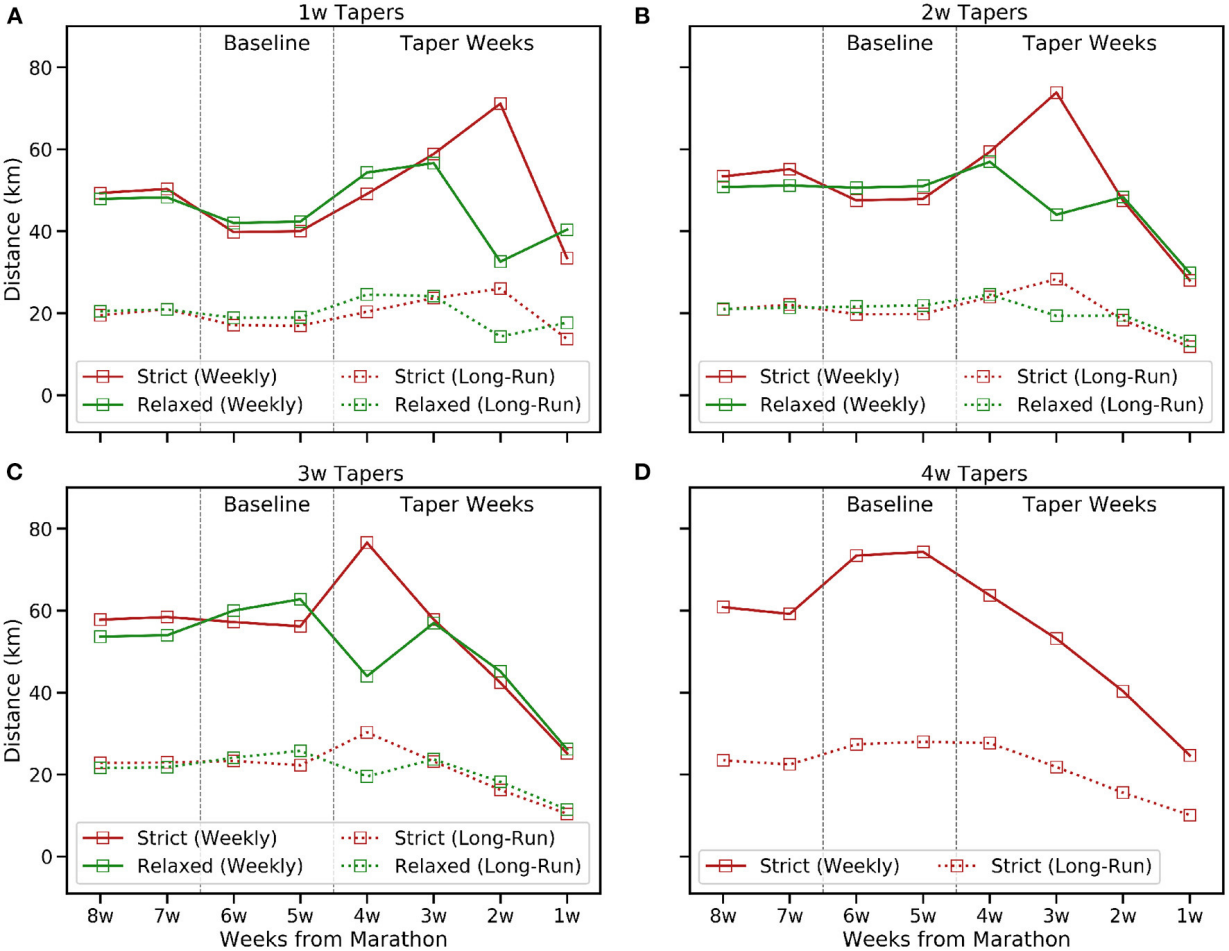
The mean weekly training distance and long-run distance (kms) in the 8 weeks before race-day for the common taper types. **(A)** 1w, **(B)** 2w, **(C)** 3w, and **(D)** 4w tapers.

**Figure 3:**
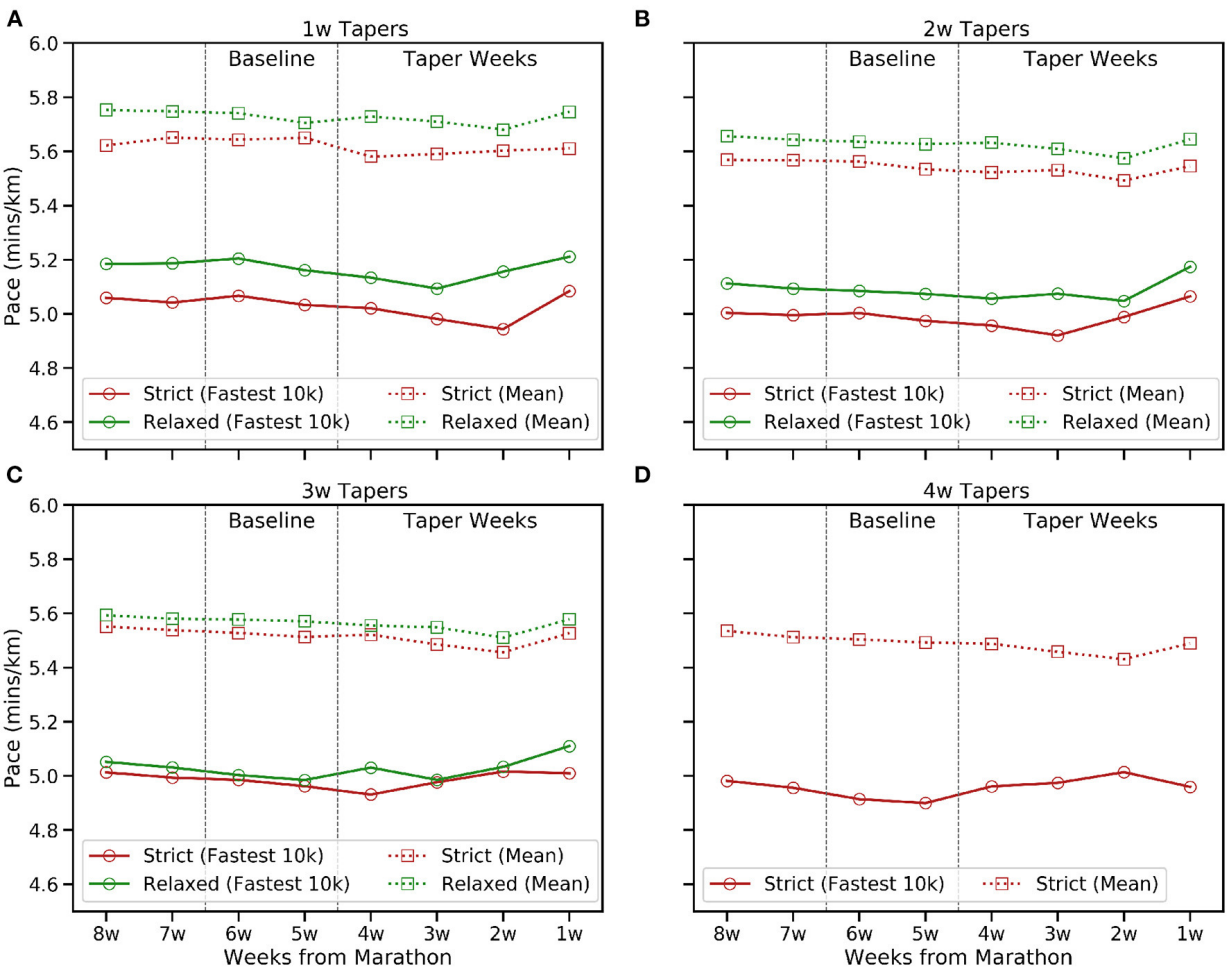
The mean weekly pace and fastest 10 km pace (min/km) in the 8 weeks before race-day for the common taper durations (1–4 weeks). **(A)** 1w, **(B)** 2w, **(C)** 3w, and **(D)** 4w tapers.

**Figure 4:**
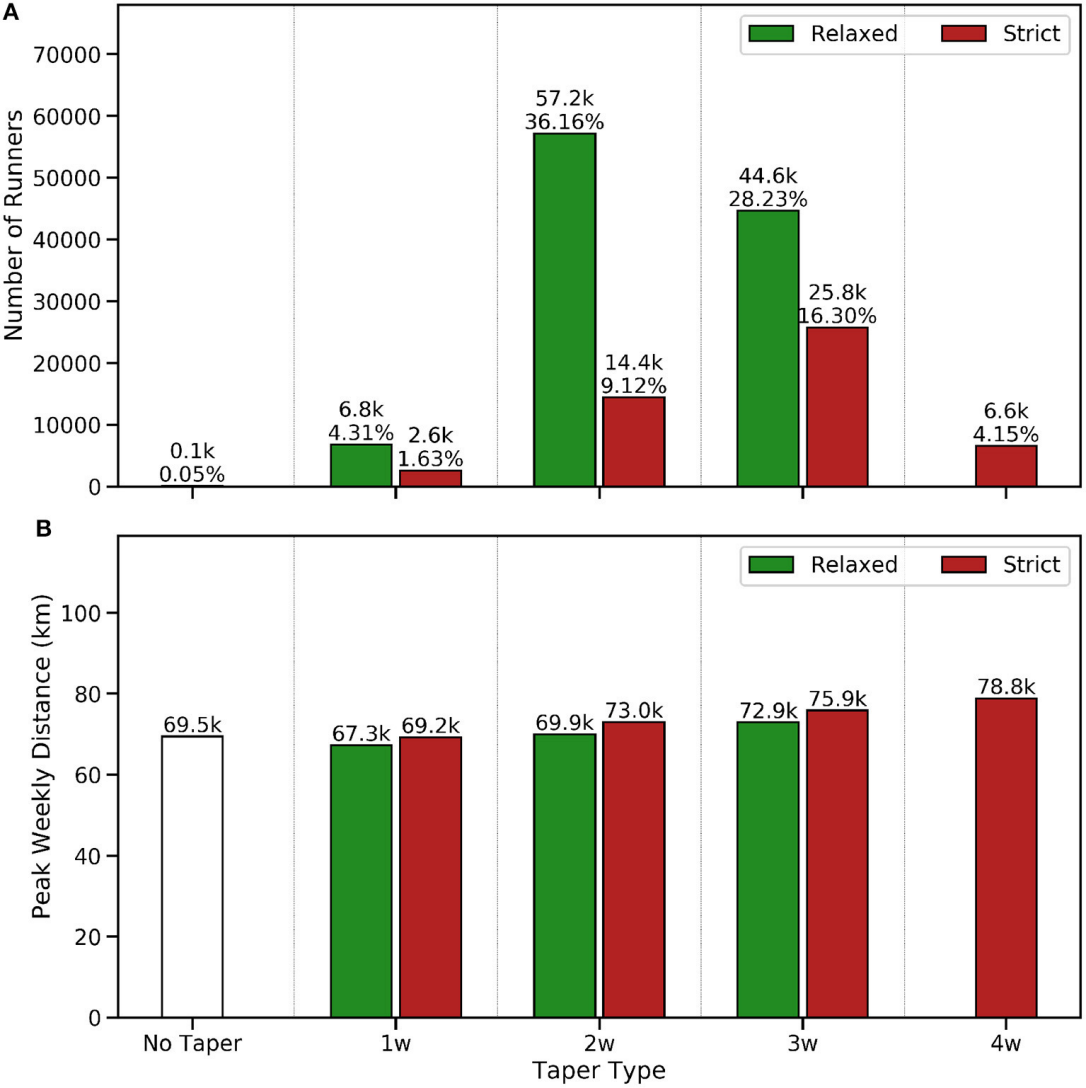
The number and percentage of runners adopting each tapering strategy **(A)** and the median peak weekly training distance **(B)** of runners by tapering strategy in the 8 weeks up to race-day.

**Figure 5:**
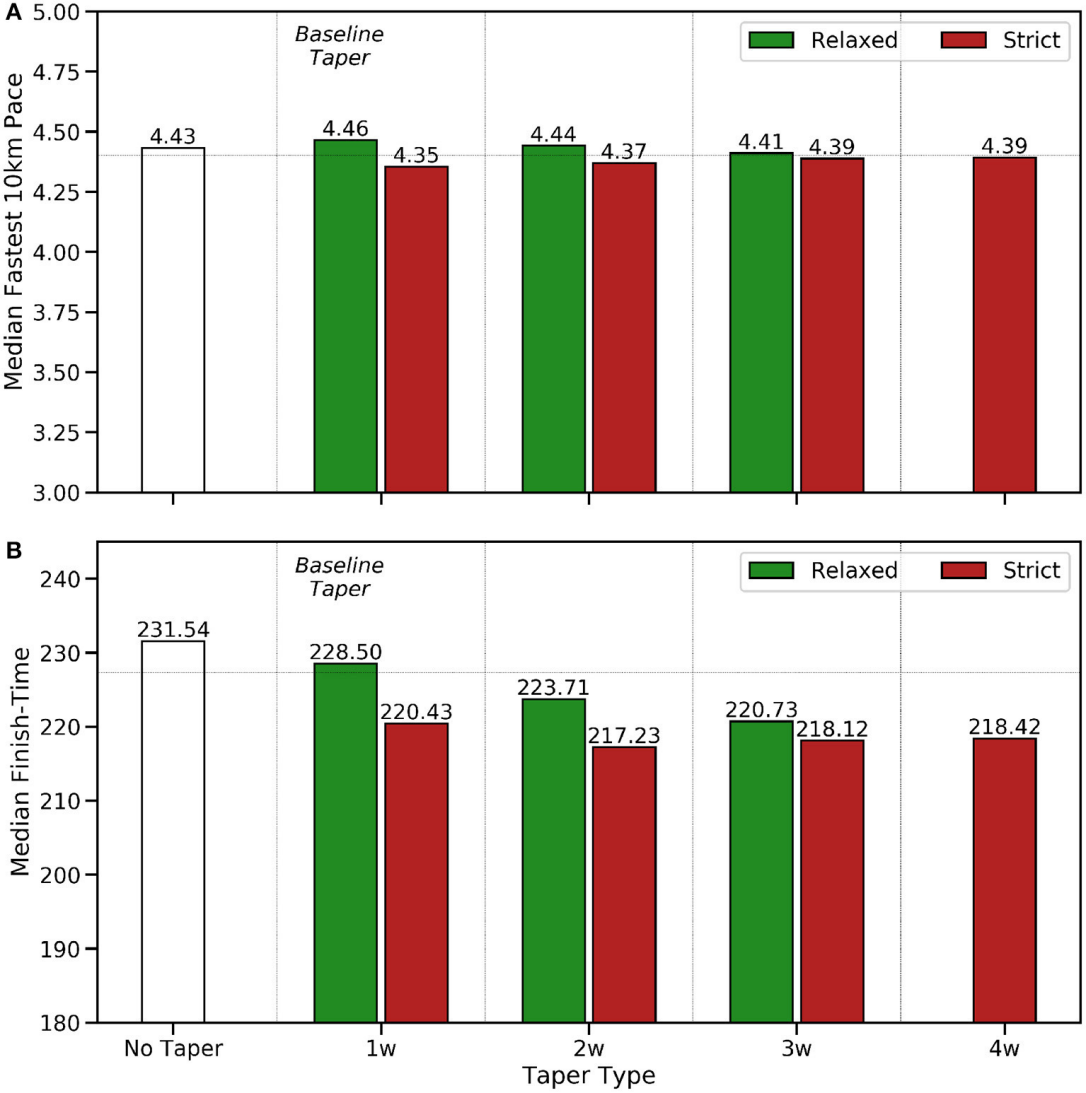
A comparison of **(A)** runner ability, based on the fastest 10 km pace (min/km) observed during training, and **(B)** marathon finish-time (FT) in minutes, by taper type.

**Figure 6:**
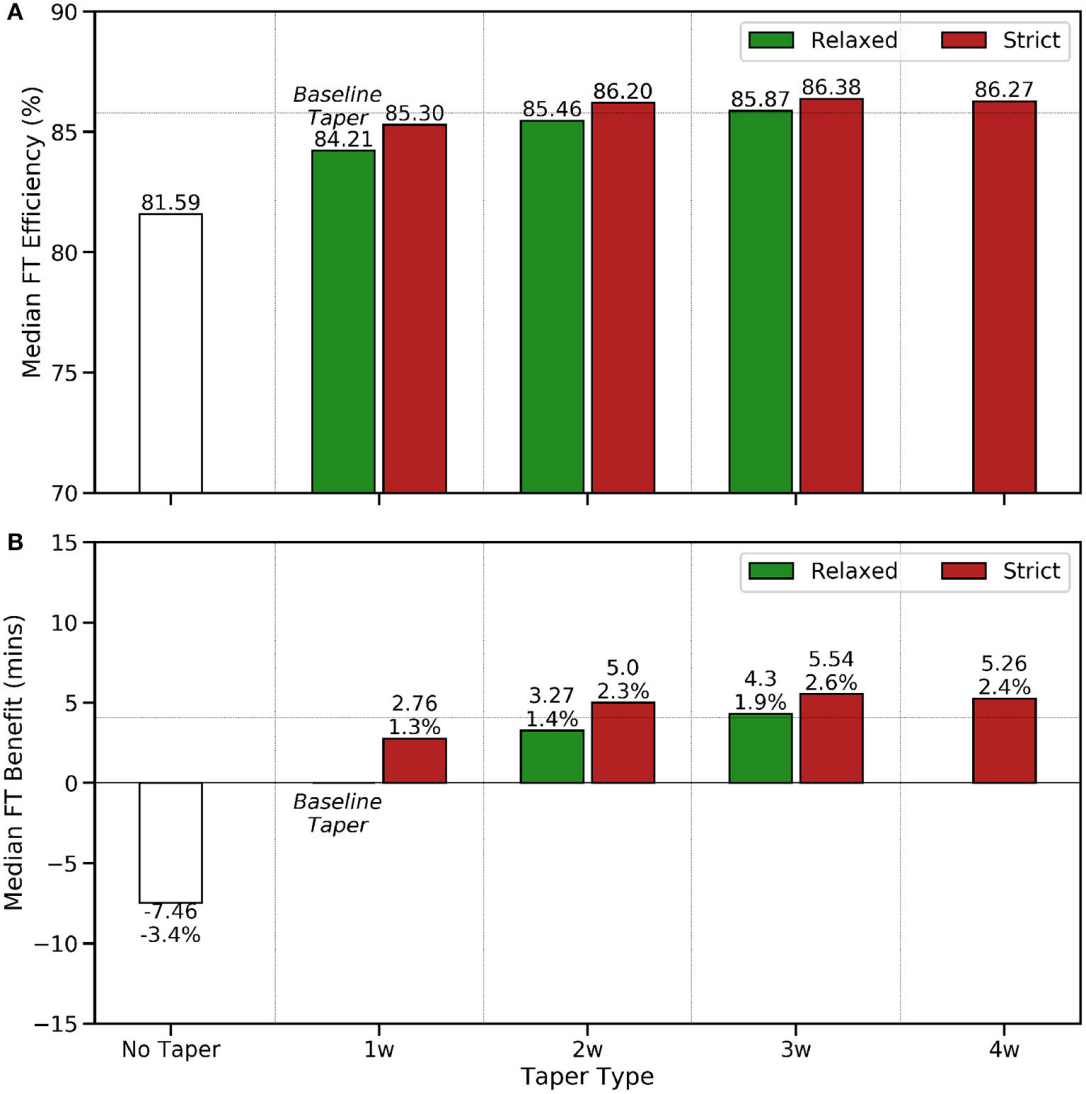
A comparison of **(A)** finish-time efficiency (FTE) and **(B)** finish-time benefit (FTB) by taper type using the relaxed 1-week taper as a baseline minimal taper.

**Figure 7:**
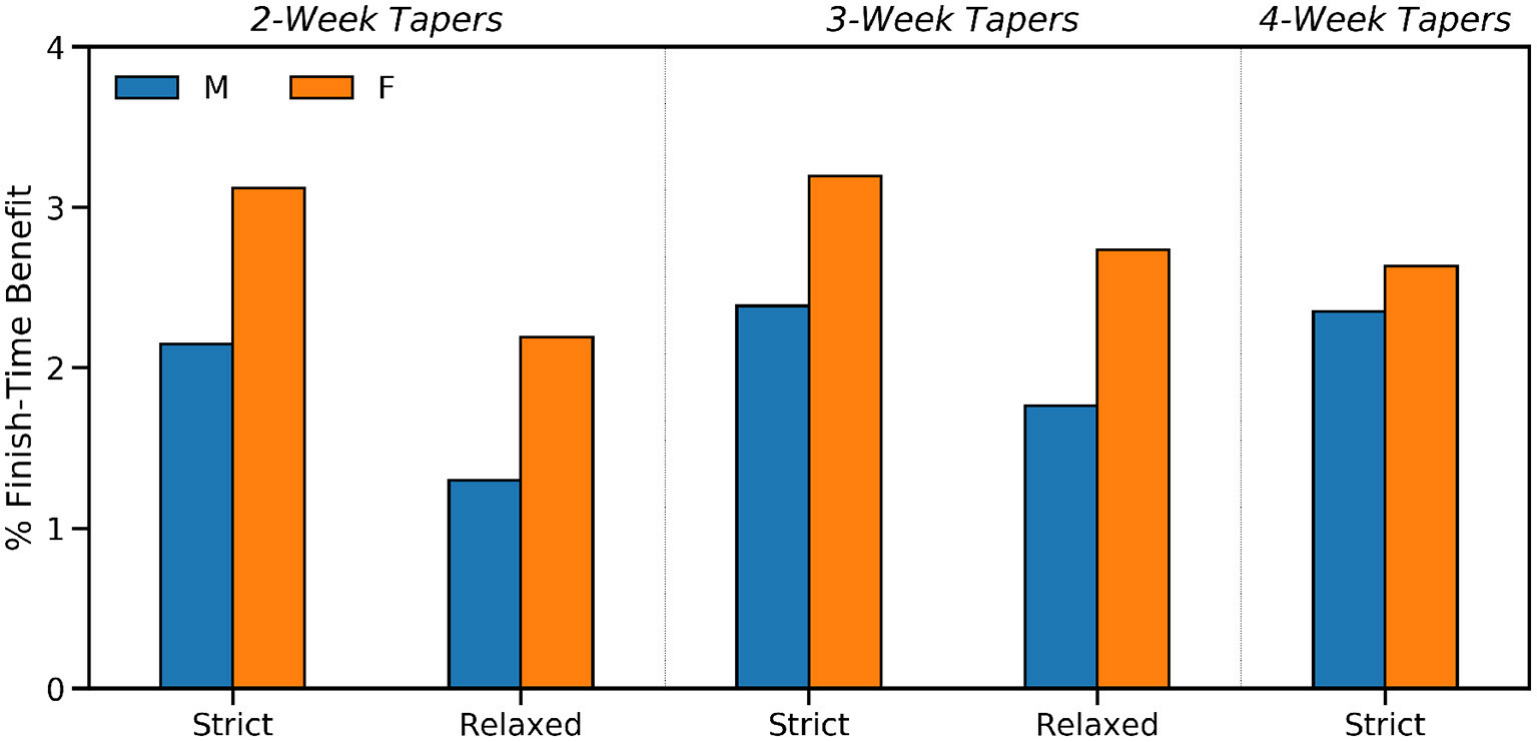
The median percentage finish-time benefit for male and female runners by taper type.

We present five sets of results as follows:

1. How activity frequency (Figure 1), weekly training distance (Figure 2), and mean weekly training pace (Figure 3) vary week by week according to taper type.
2. The adoption rates of the different taper types are presented in Figure 4A and their association with the peak weekly training distance are shown in Figure 4B.
3. The relationship between runner ability (fastest 10 km pace), marathon time, and taper type is presented in Figure 5, while the median FTE and FTB associated with each taper type are presented in Figure 6. The results of a Kruskal-Wallis test to determine the existence of statistically significant differences across these performance metrics are presented in Table 2), with posthoc Dunn's tests (using a Bonferroni adjustment) to identify which specific pairs of taper types exhibit a statistically significant difference, for *p* < 0.05, shown in Table 3.
4. The performance implications based on the sex of runners and taper type are presented in Figure 7; since the sex of a runner has a significant effect on finish-time we test this by comparing the *percentage* finish-time benefit (%FTB) for 2, 3, and 4-week tapers for male and female runners. The corresponding results of a Mann Whitney *U*-test to determine the statistical significance of the differences observed (see Table 4).
5. The results of the regression analysis to determine the relationship between different taper types and marathon performance conditioned on gender and ability are presented in Table 5.

**Table 2:** Kuskal-Wallis test results to evaluate the significance of taper type on various performance metrics: (i) marathon finish-time (FT); (ii) finish-time efficiency (FTE); and (iii) finish-time benefit (FTB).

	**DoF**	**H**	***p***
FT	7	844.18	<0.001
FTE	7	567.80	<0.001
FTB	7	521.11	<0.001

**Table 3:** The results of posthoc Dunn's tests with Bonferroni adjustment to determine which pairs of taper types show a statsitically significant effect on: (i) marathon finish-time (FT); (ii) finish-time efficiency (FTE); and (iii) finish-time benefit (FTB).

**Taper 1**	**Taper 2**	**p (FT)**	**p (FTE)**	**p (FTB)**
Strict 4 w	Strict 3 w	-	-	-
	Strict 2 w	-	-	-
	Strict 1 w	-	<0.001	<0.001
	Relaxed 3 w	<0.001	<0.001	0.002
	Relaxed 2 w	<0.001	<0.001	<0.001
	Relaxed 1 w	<0.001	<0.001	<0.001
	Non taper	-	0.003	0.007
Strict 3 w	Strict 2 w	-	-	0.035
	Strict 1 w	0.049	<0.001	<0.001
	Relaxed 3 w	<0.001	<0.001	<0.001
	Relaxed 2 w	<0.001	<0.001	<0.001
	Relaxed 1 w	<0.001	<0.001	<0.001
	Non taper	-	0.001	0.003
Relaxed 3 w	Strict 2 w	<0.001	<0.001	<0.001
	Strict 1 w	-	0.015	0.006
	Relaxed 2 w	<0.001	<0.001	<0.001
	Relaxed 1w	<0.001	<0.001	<0.001
	Non taper	-	0.020	0.038
strict 2 w	Strict 1 w	0.002	<0.001	<0.001
	Relaxed 2 w	<0.001	<0.001	<0.001
	Relaxed 1 w	<0.001	<0.001	<0.001
	Non taper	-	0.004	0.010
Relaxed 2 w	Strict 1 w	0.002	-	-
	Relaxed 1 w	<0.001	<0.001	<0.001
	Non taper	-	-	-
Strict 1 w	Relaxed 1 w	<0.001	<0.001	<0.001
	Non taper	-	-	-
Relaxed 1 w	Non taper	-	-	-

**Table 4:** The Mann Whitney U results comparing the percentage finish-time benefit of male and female runners of each taper type.

	**%M**	**%F**	**%Imp**	**U**	**p**
Strict 2 w	2.14	3.12	45.42	14,029,993.5	<0.001
Relaxed 2 w	1.29	2.19	69.05	247,103,823.0	<0.001
Strict 3 w	2.38	3.19	33.97	55,823,429.5	<0.001
Relaxed 3 w	1.76	2.73	55.32	152,948,423.5	<0.001
Strict 4 w	2.34	2.63	12.07	3,832,025.0	0.158

*The table includes the median percentage finish-time benefit for males (%M) and females (%F) with the U statistic and p value for each comparison. The column labeled %Imp shows the percentage improvement for females vs. males based on the difference between the %F and %M values*.

**Table 5:** The results of an OLS regression using the model specification in Equation 13, with dummy variables for gender and taper types, and using females and relaxed 1-week tapers as gender and taper baselines, respectively.

	**coef (β)**	**Std err**	**t**	***P*>**|**t**|****	**[0.025**	**0.975]**
*c*	–12.6241	0.525	–24.040	0.000	–13.653	–11.595
Non-taper (*taper*_1_)	0.0638	2.444	0.026	0.979	–4.727	4.855
Relaxed 2 w (*taper*_2_)	–2.3236	0.269	–8.651	0.000	–2.850	–1.797
Relaxed 3 w (*taper*_3_)	–2.9749	0.272	–10.921	0.000	–3.509	–2.441
Strict 1 w (*taper*_4_)	–2.4278	0.486	–4.998	0.000	–3.380	–1.476
Strict 2 w (*taper*_5_)	–3.6755	0.307	–11.983	0.000	–4.277	–3.074
Strict 3 w (*taper*_6_)	–4.1567	0.285	–14.598	0.000	–4.715	–3.599
Strict 4 w (*taper*_7_)	–3.6671	0.359	–10.221	0.000	–4.370	–2.964
M	4.4889	0.141	31.936	0.000	4.213	4.764
FastestPace	52.3053	0.086	611.480	0.000	52.138	52.473

*The adjusted R^2^ value for the resulting model is 0.747. The taper dummies are labeled for clarity to match the variable names used in Equation 13*.

## Discussion

### The Characteristics of Taper Types and Adoption Rates

Figures 1, 2 show how the frequency and volume of training decrease during the taper period and, generally speaking, frequency and volume peak closer to race-day for shorter tapers; this is less obvious for relaxed tapers because peaks can be spread across a multiple weeks rather than associated with a single week. In contrast, Figure 3 shows how the mean weekly pace and fastest 10 km pace are impacted in a more modest fashion; this is consistent with the common tapering advice to reduce volume while maintaining intensity. Indeed, Figure 3 shows some evidence of a speed-up in mean weekly pace during the early weeks of longer tapers as runners reduce their weekly distance by eliminating slower runs while retaining some speed-work.

Figure 4A indicates a strong preference for 2- and 3-week tapers, which account for almost 90% of all tapers, while relaxed tapers (≈69%) are more common than strict tapers (≈30%) across all durations. Only 0.05% of runners (just 84 runners) exhibit no down weeks during the taper period—the no taper group in Figure 4A—highlighting the relative rarity of these (non) tapers and suggesting such runners to be outliers. One possible explanation for this is that these runners are not actually training for their completed marathon, but are instead using the race as a stepping-stone to another event, such as an upcoming ultra-race. For completeness, Figure 4B indicates a strong positive correlation between taper duration and peak weekly distance, as runners with a higher training load in the weeks before their race employ longer tapers. In general, when we control for taper duration, then strict tapers tend to be associated with greater peak weekly distances, which could be due to more experienced runners, with greater training loads, adopting strict tapers.

### On the Performance Implications of Taper Types

In Figure 5A strict tapers are associated with faster 10 km paces than relaxed tapers and longer and more strict tapers are associated with faster marathon finish-times in Figure 5B; there is a strong correlation between the median fastest 10 km paces and marathon finish-times by taper type, which motivates the use of the finish-time efficiency (FTE) and finish-time benefit (FTB) metrics, as a way to evaluate marathon performance while controlling for ability differences.

The FTE and FTB results in Figures 6A,B indicate that longer strict tapers are associated with improved finish-time efficiencies and greater benefits. For example, strict 3- and 4-week tapers are associated with a median FTE of >86%, in comparison to just 84% for relaxed 1-week tapers, and these longer strict tapers offer runners a finish-time benefit of more than 5 min compared with the relaxed 1-week (baseline) taper. In other words, longer strict tapers are associated with runners who can complete their marathon at a pace that is closer to their fastest 10 km training pace. The implication is that shorter tapers do not permit runners to perform at this high level on race-day, regardless of their ability. And when we use the FTE of the relaxed 1-week taper as a baseline efficiency level to estimate a runner's expected finish-time—as if they had observed a relaxed 1-week taper—then we find longer and more strict tapers to offer significant finish-time benefits relative to this expected time.

The Kruskal-Wallis results in Table 2 indicate a significant effect for all three performance metrics (*H*(7)≥521.11, *p* < 0.001) and Table 3 shows the *p* values for pairs of taper types that are significantly different for *p* < 0.05. These significance results are broadly similar across the three performance metrics with the following observations noted:

1. *Strict tapers are associated with better finish-time performance (FT, FTE, FTB) than relaxed tapers for a given duration:* for *n* = 1, 2, 3, the strict form is associated with a greater performance than the relaxed form, for a given *n*, with *p* < 0.001.
2. *Longer tapers are associated with better finish-time benefits than shorter tapers:* for *n* ≤ 3, longer tapers tend to be associated with better finish-time benefits than shorter tapers (*p* < 0.05) for a given taper discipline; in fact this is usually the case regardless of discipline except for relaxed 2-week tapers compared with strict 1-week tapers where a significant FTB difference was not found.
3. *The minimal taper (relaxed 1-week) is associated with poorer performance than all other types of taper, for FT, FTE, and FTB:* the performance associated with the relaxed 1-week taper is significantly worse than all other taper types, except the *non-taper*, thereby justifying its use as a minimal taper baseline in the calculation of finish-time efficiency and benefits.

Although there is no single taper type with significantly better finish-time benefits than all of the alternatives, the strict 3-week taper offers the best all-round performance, in the sense that its finish-time benefit is significantly better than all other taper types, with the exception of the less common strict 4-week taper, and the finish-time benefits of the strict 4-week taper are not significantly different from those of the strict 3- or 2-week tapers. Broadly speaking the FTB results in Figure 6B, which indicate finish-time benefits between 1.3 and 2.4% are consistent with previous findings of a 1-3% performance benefit due to tapering (Houmard et al., 1989, 1990, 1994; McConell et al., 1993; Mujika and Padilla, 2000; Mujika et al., 2002; Bosquet et al., 2007; Luden et al., 2010; Hug et al., 2014; Spilsbury et al., 2015; Grivas, 2018; Skovgaard et al., 2018).

### Male vs. Female Tapers

Although, there is no material difference in the distribution of male and female runners by taper type, it is relevant to consider whether the sex of a runner influences performance after tapering. The results in Figure 7 indicate that females enjoy a greater median percentage finish-time benefit than males. For example, females enjoy a 3.12% median benefit for 2-week strict tapers, compared with just 2.14% for male runners, a 45% relative improvement. This pattern of improvement (shown as column *%Imp* in Table 4) is evident for each type of taper, although the difference is smaller for the 4-week tapers; it is acknowledged that these relative improvement scores, while mathematically correct, might create a misleading sense of scale in regard to these results but they are included here to help distinguish between the relative gender differences associated with the different types of taper. The *p* values for the Mann Whitney U tests confirm that all of these differences between males and females, for a given taper, are statistically significant (*p* < 0.001) with the exception of (strict) 4-week tapers. Thus, compared to a minimal taper, female runners are associated with greater finish-time benefits than males, for longer tapers up to 3 weeks (strict and relaxed).

One potential explanation for this is that it is due to pacing differences between male and female marathoners. For example, female runners have been found to be more disciplined (even) pacers (Deaner et al., 2014, 2016) whereas male runners are more likely to start too fast (Smyth, 2018) and hit the wall much more frequently (Buman et al., 2008a,b; Smyth, 2018, 2021). This may be related to a tendency among male runners to overestimate their marathon abilities compared to women (Hubble and Zhao, 2016), which may lead them to adopt more aggressive or risky pacing strategies on race-day and they are more likely to suffer the greater performance consequences if their pacing cannot be maintained.

### Cross-Sectional Regression Analysis

Table 5 shows the results of an OLS regression based on the speicifcation in Equation 13. The resulting model has an adjusted *R*^2^ of 0.747, indicating that almost 75% of the variation in marathon finish-times can be explained by the combination of gender, ability, and tapering. These results further indicate that there is a statistically significant relationship (*p* < 0.01) between each of gender, ability, all but the *non taper* type of taper (*p* = 0.979), and marathon finish-time. For example, each unit increase in a runner's fastest 10 km pace, achieved during training, leads to a 52.3 min increase in mean finish-time, all other factors remaining constant; in other words, on average a 1 min/km increase (slowing) in pace means a 52.3 min slowdown over the 42.2 km of the marathon.

Notice too how the coefficient associated with male runners (4.49) is positive, indicating that, all other things being equal, male runners are associated with finish-times that are 4.49 min longer than female runners. This does not imply that male runners are slower than females in general, but rather that, when we control for taper type and ability, then men experience a finish-time cost compared to women. This is, once again, consistent with the pacing differences that have been observed between male and female runners (Deaner et al., 2014, 2016; Smyth, 2018, 2021) and, in particular, the more disciplined, even pacing of females (Deaner et al., 2014, 2016). More evenly paced races are generally viewed to be more optimal than unevenly paced races—and certainly more desirable than the strong positive splits common among recreational male runners (Smyth, 2021)—which may explain this finish-time cost for men, and suggests that the pacing decisions made by men are leading to slower finish-times than might otherwise be achieved.

The regression results further clarify the differences between the effect of tapers on finish-time when we control for gender and ability. For all but the non-taper, we can see how tapers are associated with decreases in marathon times, relative to the relaxed 1-week taper used as the baseline in the regression, and when we control for gender and ability. For example, a strict 2-week taper is associated with a mean reduction in marathon time of 3.68 min, all other things being equal. Moreover, strict tapers and longer tapers are associated with greater finish-time reductions than relaxed tapers or shorter tapers. For example, the relaxed 2-week taper is associated with a 2.32 min finish-time reduction, compared with the 3.68 min reduction for a strict 2-week taper. And a strict 3-week taper is associated with a 4.16 min reduction compared to the 3.67 min reduction of the strict 2-week taper. The single exception is the strict 4-week taper whose finish-time reduction (3.67 min) is less than that for a strict 3-week taper (4.16 min). This is consistent with the conventional wisdom that tapers should be long enough to allow runners to recover from the accumulated fatigue of training (Morgan et al., 1987) but not so long that they begin to lose fitness, and tapers need to be carefully controlled to progressively reduce training load (Houmard et al., 1989; Mujika and Padilla, 2000).

### Implications for Recreational Runners

Given that strict tapers are associated with improved performance, and that only 31% of recreational runners adopt strict tapers, then there is an opportunity for many recreational runners to improve their marathon performance by simply switching to this more disciplined taper format. In many cases this could be as straightforward as re-sequencing their taper weeks to implement a more consistent decrease in training volume. Indeed, the results in Figure 7 suggest that by switching from a 2/3-week relaxed taper to a corresponding strict taper, runners could improve their percentage finish-time benefits considerably. For example, a switch from a relaxed 2-week taper to a strict 2-week taper is associated with an improvement for males from 1.29% (relaxed) to 2.14% (strict) and a corresponding improvement for females from 2.19 to 3.12%; the scale of the improvement is less for 3-week tapers but still material (1.76–2.38% for men and 2.73–3.19% for women). This reduced improvement for 3-week tapers is likely due to the fact that there are more possible combinations of low quality, relaxed 2-week tapers than there are for 3-week tapers. By definition, a relaxed 3-week taper can only accommodate a single up week between its down weeks—it must involve at least two consecutive down weeks—whereas some relaxed 2-week tapers will include runners with two consecutive up weeks, perhaps directly before race-day. Thus, we can expect a greater opportunity for improvement when moving from a 2-week relaxed taper to a 2-week strict taper, than when moving from a 3-week relaxed taper to a 3-week strict taper.

The regression analysis also supports the view that recreational male runners tend to make sub-optimal pacing decisions that adversely impact their marathon performance; this is expressed as a finish-time cost for males when we control for taper type and ability. Thus, male runners should not only consider their tapering strategy but also their race-day pacing if they wish to optimize their race-day performance; men are forgoing 4.49 min on average when we control for ability and tapering because they tend to start faster and pace less evenly than women.

### Limitations

As more and more runners routinely track their activities using mobile devices and sensors, it is increasingly feasible to conduct new types of data-driven research to better understand how people train and perform. The scale of the data sets that can be generated may overcome some of the limitations of more traditional, small-scale, selective studies. We believe that this has the potential to help sports scientists to produce more robust conclusions and could help exercise physiologists to produce more actionable insights for coaches and athletes. This research is one such example of the type of study that can be conducted at scale, but there are a number of methodological considerations and limitations that need to be acknowledged.

First, the data set used is based on raw activity data collected by a popular fitness application. It has been minimally cleaned, anonymised, and processed to extract 100 m pacing data, as discussed, but it has not been validated for individual runners. While there are sufficient data to be confident about trends observed and the associations implied, it is also true that many of the factors that likely impact marathon training and performance (injury, weather, desire, competitiveness etc.) are absent. For example, if a runner has a strong desire to achieve a personal-best time or to finish within an important landmark time (3 or 4 h for example), then this may impact performance (Allen et al., 2017; Markle et al., 2018) and taper discipline. Similarly, groups of runners who train together might be more likely to taper in a similar way. Certainly, the circumstances of a given race and race day will impact performance: the topology of the course, weather conditions (Montain et al., 2007; Ely et al., 2008; Marr and Ely, 2010; Vihma, 2010; Guo and Fu, 2019; Knechtle et al., 2019), crowd support (Russell, 1983; Epting et al., 2011), the level of competition (Corbett et al., 2012), even the time of day (Capp, 1999; Fernandes et al., 2014) have all been shown to impact athletic performance. Unfortunately, it has not been possible to consider these factors in the present study because they are absent from our data set and the anonymisation procedure has further obfuscated features than might be used to single out an individual runner. This means that demographic features, such as age, and even race identifiers have been removed. As a matter of future work it is hoped that some of these limitations may be overcome to accommodate a more in-depth analysis by catering for the fixed effects of runners and races using a regression analysis.

Another consideration is the use of the fastest 10 km pace observed during training as a proxy for a runner's ability and its subsequent use in the estimation of finish-time benefits; a related approach was adopted by Zrenner et al. (2021). It is not possible to verify whether an observed fastest 10 km pace is accurate for a given runner because it depends very much on the style of their training. The 10 km distance was chosen because most runners, while training for a marathon, are likely to participate in some shorter distance time-trials or races, making 10 km a reasonable target distance to used as a proxy for ability. However, the fastest 10 km pace estimate will likely underestimate a runner's true ability if they are disinclined to perform maximal effort training sessions, but since this could also be the case in their marathon, the relationship between their marathon pace and their fastest 10 km pace could still serve as a useful estimate of finish-time efficiency and, ultimately, finish-time benefit. Regardless, estimating runner ability is one area for improvement in this work. For example, one option may be to use a more robust estimate of runner ability such as the *critical speed*, which can be estimated using raw training data (Smyth and Muniz-Pumares, 2020).

There is an obvious selection bias in the construction of the data set used because for each runner only their fastest marathon in a given year is included. The reason for this decision is that while some runners did complete more than one marathon per year, it was usually the case they were targeting a particular marathon as their primary goal-race and, as such, one could expect their training and tapering to differ for their slower races. Were we to include all of the marathons for a given runner then it could produce overlapping training data sets, if multiple marathons occurred within 23 weeks of each other. One consequence of the decision to focus on the fastest races each year is that it could over estimate the effect of tapering on race performance. However, it is worth noting that only 34% of runners registered more than one marathon in a given year and thus in a majority of cases there was only a single marathon to consider. In any case, it is more correct to view the analysis as presented as one that compares the tapering strategies of runners for their fastest marathons in a given year.

In this work we have compared different taper types to a 1-week relaxed taper as a nominated control. However, it was not possible to provide a control on a runner-by-runner basis, because many runners have completed only a single marathon within the time-frame of the data set, and those that have completed more than one marathon often do not vary their taper approach across multiple races and years. Thus, although the results indicate that longer and more disciplined tapers are associated with improved race performance, we cannot be certain that this will be case for every runner if they change from a 1-week relaxed taper to a longer or more disciplined taper. Nevertheless, the performance differences observed for the different taper types are generally highly significant (*p* < 0.001) and unlikely to have occurred by chance.

## Conclusions

In this study, we used a large data set of raw training and race data from recreational marathon runners to evaluate their different tapering strategies in the weeks before race-day, and their resulting performance on race-day. We proposed a novel framework for comparing the different types of tapers implemented by recreational runners. We found that longer tapers and more disciplined (strict) tapers were associated with improved performance benefits for recreational runners and that these benefits were greater for female runners than for male runners. Although a large majority of recreational runners (≈90%) tended to favor a 2- or 3-week tapers, most runners (≈69%) adopted less disciplined forms of taper. An important practical implication of this work is that there could be an opportunity for many runners to improve their relative performance by implementing a more disciplined form of taper. This is likely to be of considerable interest to recreational marathoners and coaches.

## Data Availability Statement

The data analyzed in this study is subject to the following licenses/restrictions: The anonymous data used in this study was made available to the authors as part of a limited data sharing agreement with Strava Inc. Requests to access these datasets should be directed to http://strava.com.

## Ethics Statement

The studies involving human participants were reviewed and approved by Human Research Ethics Committee (Sciences) at University College Dublin. Written informed consent for participation was not required for this study in accordance with the national legislation and the institutional requirements.

## Author Contributions

BS and AL contributed to conception and design of the study and contributed to manuscript revision, read, and approved the submitted version. AL organized the database and performed the data collection and cleaning. BS performed the statistical analysis and wrote the first draft of the manuscript. All authors contributed to the article and approved the submitted version.

## Funding

This work is supported by Science Foundation Ireland under grant 12/RC/2289P2 with data provided by Strava Inc. as part of a data sharing agreement. Science Foundation Ireland and Strava had no role in study design, data collection and analysis, or the preparation of this manuscript.

## Conflict of Interest

The authors declare that the research was conducted in the absence of any commercial or financial relationships that could be construed as a potential conflict of interest.

## Publisher's Note

All claims expressed in this article are solely those of the authors and do not necessarily represent those of their affiliated organizations, or those of the publisher, the editors and the reviewers. Any product that may be evaluated in this article, or claim that may be made by its manufacturer, is not guaranteed or endorsed by the publisher.

## Footnotes

^1^ https://www.strava.com

^2^ https://www.runkeeper.com

^3^ https://www.mapmyrun.com
